# The Role of Cyclooxygenase in Multiplication and Reactivation of HSV-1 in Vestibular Ganglion Neurons

**DOI:** 10.1155/2014/912640

**Published:** 2014-02-05

**Authors:** Yuehong Liu, Shufeng Li, Zhengmin Wang

**Affiliations:** Department of Otology and Skull Base Surgery, Eye, Ear, Nose, & Throat Hospital of Fudan University, 83 Fenyang Road, Shanghai 200031, China

## Abstract

Reactivation of latent herpes simplex type 1 (HSV-1) and nerve inflammation have been shown to be involved in vertigo-related vestibular pathogenesis. Treatments of such diseases have been less than perfect. Nonsteroidal anti-inflammatory drugs (NSAIDs) have been reported to suppress reactivation of HSV-1 in trigeminal ganglions. However, whether this drug can affect reactivation of HSV-1 in vestibular ganglions is unclear. Due to the difficulties of constructing *in vivo* animal models, in this study, we developed a vestibular ganglion culture system, in which vestibular neurons were latently or lytically infected with HSV-1. Indomethacin and celecoxib were selected to measure their effects on HSV-1. Trichostatin A was used to reactivate HSV-1 in latently infected neurons. Cycloxygenase-2, which is the target of NSAIDs, was induced by HSV-1 in the lytically infected cultures, with an increase of 14-fold. Although it appeared that indomethacin and celecoxib showed limited but concentration-dependent inhibition effects on viral production under our condition, indomethacin decreased reactivation rate of HSV-1 by about 20%. Though more *in vitro* or *in vivo* studies are needed to confirm the effects of the drugs, our study may provide a potential way to investigate the mechanism of HSV-related vestibular pathogenesis as well as new treatments of vertigo-related diseases.

## 1. Introduction

Intermittent vertigo is considered to be one of the most debilitating symptoms in clinical work. Meniere's disease (MD), benign paroxysmal positional vertigo (BPPV), and vestibular neuronitis (VN) are the most common clinical syndromes that manifest as recurrent vertigo. The recurrent nature of this dysfunction implies a reversible alternation in vestibular nerve physiological function caused by changes in the neuron or in its environment. Etiologies of these vertigo-related diseases remain largely unknown. Cumulative evidence has suggested primary vestibular nerve inflammation by viral agents in MD and BPPV [1–4]. Immunological evidence also supports the presence of neurotropic (NT) virus in such patients [5]. Herpes simplex virus-1 (HSV-1) DNA or HSV latency-associated transcripts (LAT), the latter of which is the only transcript produced during latent infection, have been detected in the vestibular ganglia (VG) surgically excised from MD patients [6, 7]. Meanwhile, VN has been most commonly hypothesized to be the result of an infection of the vestibular nerve by HSV-1 [8]. HSV-1 DNA has been amplified in the vestibular nuclei of patients with a history of VN [9]. These evidences support the concept of intermittent reactivation of latent NT virus in the VG and the pathogenesis of fluctuation of clinical symptoms [2, 3]. However, poor progress has been made in the field of HSV-related vestibular pathogenesis in recent years, which is mainly caused by the lack of animal models. Due to the fact that VG is deeply imbedded in the temporal bone, incidence of virus transmitting to the VG in animal models is rather low and with high mortality [10, 11]. Fortunately, recent researches indicated that HSV-1 lytic and quiescent infection can be established in primary neuronal cell cultures of sympathetic ganglia and VG [12, 13]. Latent infection of HSV in these neurons can be reactivated by addition of certain drugs or withdrawal of some nutrition factors from the culture medium. These might shed a light on the research of HSV-related vestibular pathogenesis.

Interestingly, we accidentally found that indomethacin, one of nonsteroidal anti-inflammatory drugs (NSAIDs), has some therapeutic effects on the acute attacks of MD (data not shown here). NSAIDs can pharmacologically target cyclooxygenase (COX) isozymes, COX-1 and COX-2, finally inhibiting the expression of prostaglandin (PG), which is an important proinflammatory mediator of inflammatory response. Previous studies have shown that NSAIDs were able to suppress HSV reactivation in murine trigeminal ganglions (TGs) [14, 15], as well as inhibit the multiplication of some other types of virus in cell lines [16, 17]. In light of these, we suppose that COX might intensify attacks of MD through promoting viral production or enhancing the severity of nerve inflammation. And NSAIDs would take positive effects through either inhibiting the production and reactivation of HSV-1 or reducing viral neuroinflammation in VG. Animal models or cell culture models of viral infection and routes of drug administration targeted at vestibular ganglia therefore would be important tools to understand the pharmacotherapy of NSAIDs. Thus, for the first time, we developed a cell culture model system to measure the COX induction level upon HSV-1 infection and to investigate the effects of NSAIDs on HSV-1 replication and reactivation in vestibular ganglion neurons (VGNs).

## 2. Materials and Methods

### 2.1. Herpes Virus Stock Preparation

HSV-1 virus, GHSV-UL46, in which GFP was incorporated into the tegument protein VP11/12 (which served as the reporter protein), was purchased from American Type Culture Collection (ATCC). The expression and incorporation of the recombinant protein do not significantly alter virus development [18]. HSV-1 stocks were grown from Vero cell cultures infected at low MOI. Viral titers were obtained using TCID50 of serial dilutions on cultured Vero cells.

### 2.2. VGN Purification and Cell Culture

The following procedure was approved by the Ethics Review Board of our institute. Following administration of anesthesia, vestibular ganglia were harvested from 5-day-old Sprague-Dawley rat pups. These ganglia were treated with 0.025% trypsin and 0.02% collagenase (Sigma, St. Louis, MO, USA) for 30 minutes at 37°C and then were triturated, filtered through a 70 *μ*m filter (BD Falcon, Franklin Lakes, NY, USA). The cells were plated on 96-well plates (BD Falcon) coated with poly-L-lysine (0.01%; Sigma) and laminin (20 *μ*g/*μ*L; Sigma). The same number of neuronal and nonneuronal cells was plated in each well. Cell culture media consisted of neurobasal media (NBM; Invitrogen, Grand Island, NY, USA) supplemented with B27 (Invitrogen), 5% FBS (Invitrogen), and ampicillin (0.1 mg/mL; Invitrogen). To kill rapidly dividing cells, the cultures were treated with 20 *μ*mol/L 5-fluoro-2′-deoxyuridine (5-FU; Sigma) and 10 *μ*mol/L aphidicolin (Sigma) and were serum starved for the first 2 days as described in the previous article [12].

### 2.3. HSV-1 Infection and Drug Treatment

Induction of primary lytic or latent HSV-1 infection was performed as previously described [12]. On day 4 when the neurons were cultured *in vitro* (DIV 4), one hour prior to infection with HSV-1, the cultures were treated either with inhibitors of prostaglandin synthesis or exogenous prostaglandin E_2_ (PGE_2_) (Sigma). Inhibitors of indomethacin (Sigma) and celecoxib (Sigma) were dissolved in dimethyl sulfoxide (DMSO) to a stock solution of 200 mmol/L and 25 mmol/L, respectively, and were diluted to working concentrations with fresh medium. PGE_2_ was dissolved in ethanol to 5 mg/mL and was further diluted with fresh medium. A solvent control of 0.25% DMSO was used in experiments whenever indomethacin or celecoxib was used. VGNs were exposed to HSV-1 at a MOI of 0.01 for 1.5 hours. Then, medium was replaced with fresh cell culture medium (NBM/B27/FBS/ampicillin) containing either drugs or DMSO. A multichannel pipette was used to ensure that the exact same volume of culture medium was added to each well. Cultures infected by HSV-1 remained in the drugs for 48 hours, and the cells and supernatants were collected to measure the titers of virus. The drug cytotoxicities were determined using the Cell Titer Aqueous One solution (Promega, Madison, MI, USA). All of the inhibitors were used at concentrations that were not toxic to the cells.

Establishment of latent HSV-1 infection was achieved by treating the neurons with 100 *μ*mol/L acyclovir (ACV; sigma) at DIV 2. At DIV4, the neurons were exposed to HSV-1 in the presence of ACV. After the virus was removed, cultures continuously remained in the ACV for 4 days. At DIV 8, the medium with ACV were replaced with fresh medium containing either 100 *μ*mol/L indomethacin or DMSO one hour before 1 *μ*mol/L trichostatin A (TSA; Sigma) was added into the medium to induce reactivation. TSA-containing media was left in the cultures for 30 minutes at 37°C and then replaced with media without TSA but containing 100 *μ*mol/L indomethacin. Indomethacin was also used in the baseline controls (media changed but no TSA added) to determine the effect on baseline reactivation. The neurons were examined daily for the presence of GFP-positive wells using fluorescence microscopy (Zeiss Axiovert 200M, USA). The wells with GFP-positive cells prior to ACV removal were not used to determine the rates of viral reactivation.

### 2.4. Immunofluorescence

VGNs were plated on glass slides and fixed with 4% paraformaldehyde in PBS for 2 hours at 4°C, permeabilized with 0.2% Triton X-100. Mouse antineurofilament 200 (NF200) (1 : 500, Sigma) and rabbit anti-COX-1/COX-2 (1 : 300, Abcam) were added to the cells at 4°C overnight. Then cells were incubated in Rhod (TRITC) AffiniPure donkey anti-rabbit or Alexa Fluor 488 donkey anti-mouse or Rhod AffiniPure donkey anti-mouse secondary antibody (1 : 500, Invitrogen) for 1 hour at 37°C and were counterstained with DAPI (1 : 800, Invitrogen) for 15 minutes to stain nuclei prior to final embedding. Fluorescent images were obtained using a NIKON H600L microscope (Japan).

### 2.5. Reverse-Transcription Polymerase Chain Reaction (RT-PCR) and Real-Time PCR

4 days after lytic infection, quiescent infection, and mock infection, cells were harvested in Trizol (Invitrogen). Total RNA was extracted from 6 wells of lytically infected cells with HSV-1 (per condition), 4 wells of latently infected cells, and 4 wells of mock-infected cells. The cDNA was synthesized using Superscript III Reverse Transcriptase (Promegah). PCR analysis of LAT, ICP27, and COX-1/COX-2 was performed using PCR reagents (TaKaRa, Tokyo, Japan). The LAT and ICP27 primer sequences have been previously described by Camarena et al. [13]. COX primer sequences were the following: COX-1: F5′ctatcactggcatccgttcat3′, R5′aaagttcctacccccaccaat3′; COX-2: F5′ggccatggagtggacttaaa3′, R5′acgtggggagggtagatcat 3′. Real-time PCR reaction mixtures were prepared using SYBR Green (TaKaRa) and run on the StepOne Plus Real-time PCR System (Applied Biosystems) using the following program: 95°C for 5 minutes, 95°C for 5 seconds, and 60°C for 30 seconds, for 40 cycles. The results were analyzed using the ddCt method, and the experiments were performed in technical triplicates and repeated independently at least twice.

### 2.6. Western Blotting Analysis

Per condition, protein extracts from 16 wells of VGN cultures were separated by sodium dodecyl sulfate on a 10% Tris-HCl minigel (Bio-Rad, Hercules, CA, USA). The gels were then transferred onto polyvinylidene fluoride (PVDF) membranes (Millipore, Billerica, MA, USA). Membranes were probed for the rabbit polyclonal COX-1 and COX-2 primary antibodies (1 : 1000; Abcam) overnight at 4°C. After several washes, these membranes were incubated with HRP-conjugated goat anti-rabbit IgG (1 : 5000, Abmart, Shanghai) for 2 hours at room temperature and were incubated with enhanced chemiluminescence (ECL, Beyotime, Shanghai) reagent for 1 minute, and finally was exposed to film and developed using an SRX-101A film processor (Konica Minolta Medical and Graphic Inc., NJ, USA).

### 2.7. Viral Titer Determination

At the indicated times after-infection, the VGN cultures treated with or without drugs were collected and frozen at −80°C. After three cycles of freezing and thawing, the samples were titrated using TCID50 to determine the virus yields.

## 3. Results

### 3.1. COX-1/COX-2 Are Constitutively Expressed by VGNs

**Figure 1:**
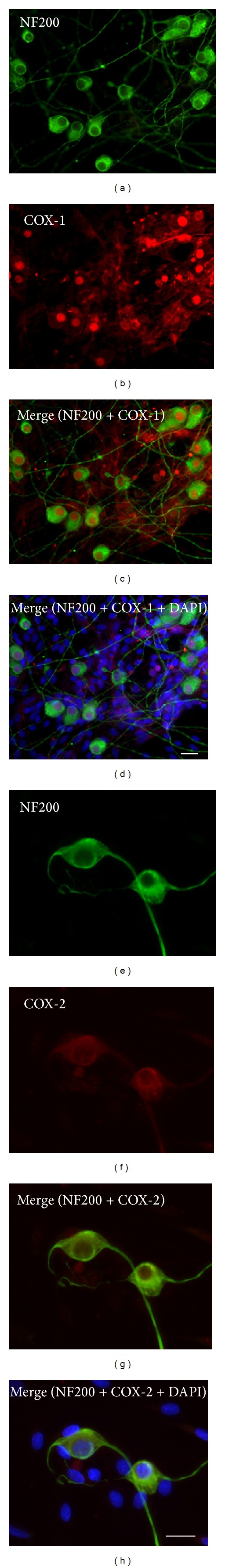
Immunofluorescence of COX-1 and COX-2 in VGNs. All of the neurons identified as NF200-positive (a) expressed COX-1 in either the cytoplasm or nuclei (b) and had an increased signal in the nuclei as confirmed by the merged images ((c), (d)). In comparison, weak COX-2-specific staining was identified mainly in the cytoplasm of VGNs as shown in ((e), (f)) and was confirmed by the merged images ((g), (h)). Scale bar, 25 microns.

COX-1 has been proposed to be expressed constitutively, while COX-2 enzyme is primarily an inducible enzyme. In the present study, double-immunofluorescence labeling showed a clear cytoplasmic and nuclear COX-1 enzymatic immunoreaction in VGNs, which were more intensely labeled in the nuclei. Nonneuronal cells were also COX-1 immunopositive, but with less intensity (Figures 1(a), 1(b), 1(c), and 1(d)). Furthermore, COX-2 enzyme was identified mainly in the cytoplasm of VGNs (Figures 1(e), 1(f), 1(g), and 1(h)). However, the immunofluorescent reaction to COX-2 in the nonneuronal cells, including Schwann cells and fibroblasts, was not robust.

### 3.2. COX-1/COX-2 mRNA and Protein Levels of VGN Cultures Increase upon HSV-1 Infection

**Figure 2:**
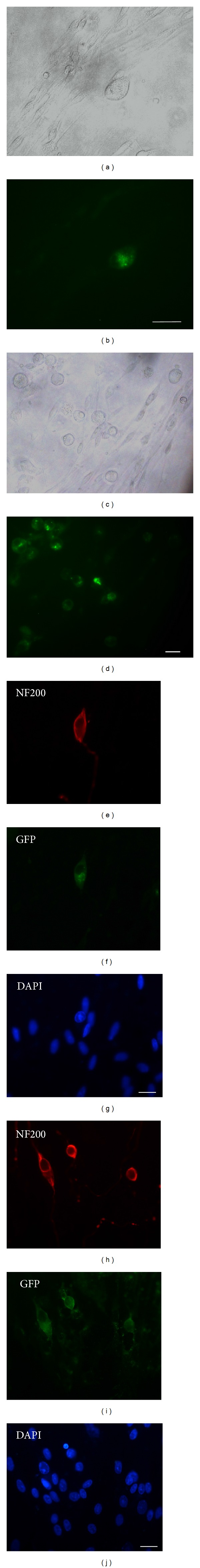
Pattern of HSV-1 infection in VGN cultures. VGN cultures following lytic infection with HSV-1/UL46-GFP using phase contrast microscopy ((a), (c)) and fluorescent photomicrographs ((b), (d)). Double labeling of intrinsic GFP fluorescence ((f), (i)) and NF200 immunostaining of neurons ((e), (h)). Scale bar, 20 microns.

**Figure 3:**
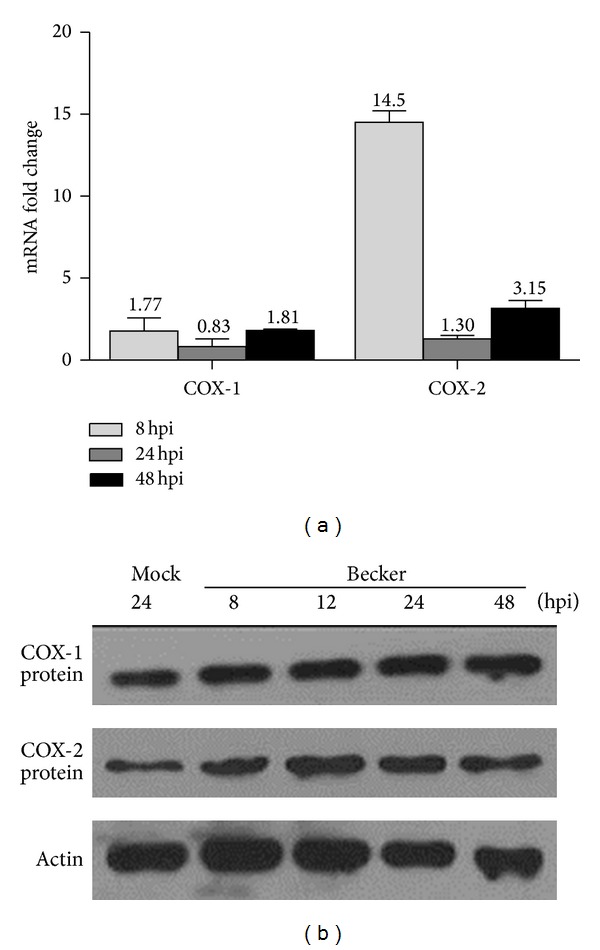
HSV-1 infection induces COX-2 mRNA and protein expression. (a) The numbers show the precise fold changes at indicated times after infection with COX-1 or COX-2-specific primers. (b) Western blotting shows the expression pattern of COX-1/COX-2 following HSV-1 infection at indicated time points (top two panels).

Having demonstrated the localization of COX-1/COX-2 enzyme in VGNs, we next examined the induction of cyclooxygenase mRNA by performing real-time PCR with cDNAs obtained from cultures at indicated times after infection. Given that HSV-1 can cause inflammatory infiltration in other types of trigeminal ganglia [19], it is possible that cyclooxygenase, which is a type of proinflammatory factor, could be induced by the virus in our model. At a low multiplicity of 0.01, virus initially infected the neurons, which was confirmed by identifying the GFP-positive cells under fluorescent microscopy (Figure 2). Thus, HSV-1 might initially modulate the cellular gene expression of vestibular neurons. According to our data (Figure 3(a)), relatively little COX-2 mRNA was detected in the mock-infected cells. However, after infection, an increased amount of mRNA was evident at 8 hours after infection (hpi), with an increase of 14-fold. Less COX-2 mRNA was present at 24 hpi, at which point the mRNA levels had nearly returned to basal levels. However, at 48 hpi, the mRNA levels were slightly elevated, an effect that might be due to the spread of the virus to adjacent nonneuronal cells. The transcript level of COX-1 indicated a slight (less than 2-fold) induction of this transcript upon HSV-1 infection. Western blotting analysis (Figure 3(b)) showed that COX-2 protein was induced, whereas the level of COX-1 protein was less affected. Although COX-2 mRNA levels were transiently induced, COX-2 protein levels remained substantially induced at 48 hpi. Thus, the protein has a longer half-life compared to the mRNA.

### 3.3. COX Inhibitors Showed Limited but Concentration-Dependent Inhibition Effect on the Production of Infectious HSV-1 Progeny in VGN Cultures

At DIV 4, the VGN cultures were exposed to HSV-1/GFP at a low multiplicity of infection (MOI). Under continuous observation using fluorescent microscopy, typically the neurons first expressed GFP in each well at 24 hpi, with adjacent cells becoming GFP-positive by 48 hpi, and exhibited typical cytopathological features of HSV-1 infection as assessed by differential interface contrast (Figures 2(a), 2(b), 2(c), and 2(d)). This progression pattern was confirmed by double labeling with intrinsic GFP fluorescence and immunostaining with NF200 (Figures 2(e), 2(f), 2(g), 2(h), 2(i), and 2(j)) under fluorescent microscopy. GFP expression in the cytoplasm of VGNs was more intense compared with adjacent cells. As COX protein was induced during the first 48 hpi, we chose the first 48 hours of HSV-1 infection as the time point to investigate the effects of COX inhibitors on virus spread and propagation.

**Figure 4:**
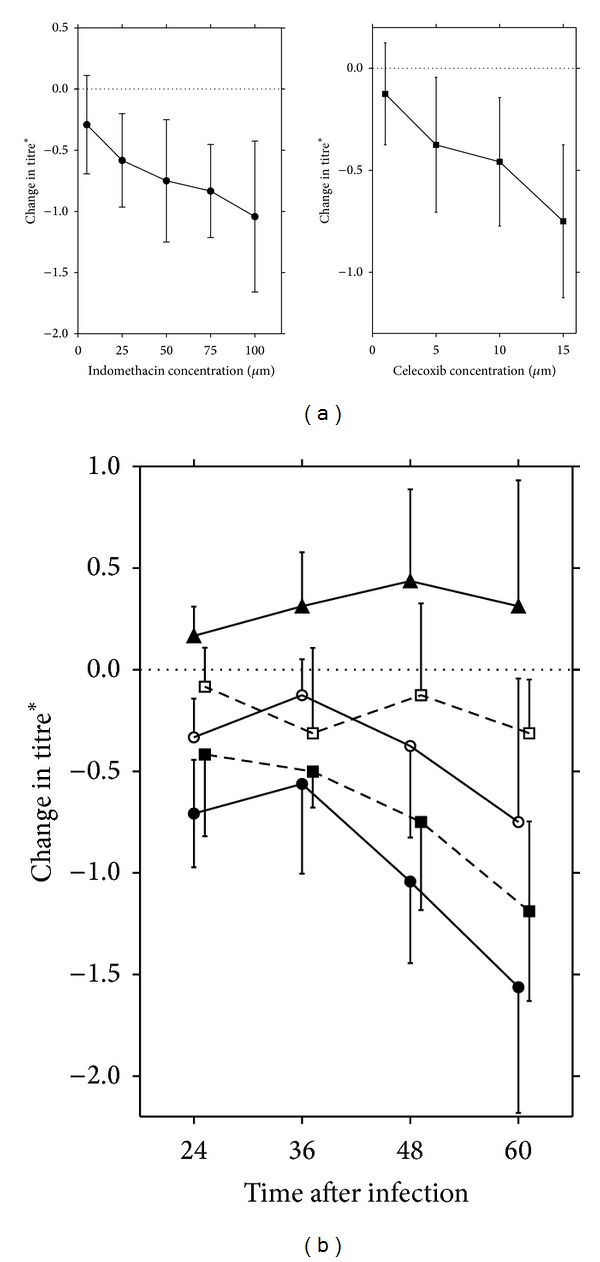
The effects of inhibitors of prostaglandin synthesis and PGE_2_ on the yields of HSV-1 in VGN cultures. (a) The cultures were collected and titrated at 48 hpi. (b) ●, 100 *μ*mol/L indomethacin; ○, 100 *μ*mol/L indomethacin + 15 *μ*mol/L PGE_2_; ▲, 15 *μ*mol/L PGE_2_; ■, 15 *μ*mol/L celecoxib; □ 15 *μ*mol/L celecoxib + 15 *μ*mol/L PGE_2_. *Log geometric mean TCID50 with drug minus log geometric mean TCID50 without drug.

We used two COX inhibitors in our studies: indomethacin, a nonspecific inhibitor of both cyclooxygenase isoforms; and celecoxib, a COX-2-specific inhibitor. Using TCID50, we tested increasing concentrations of each inhibitor for their effects on virus yield at 48 hpi in HSV-1 infected VGN cultures. Each time point represents the mean yield obtained from 2 to 3 experiments. The bars represent the standard deviations between experiments. Under our condition, both inhibitors showed limited inhibition effects on HSV-1 growth, which appeared to be concentration-dependent (Figure 4(a)). Indomethacin (100 *μ*mol/L) decreased yields, in this case by approximately 10-fold at 48 hpi (Figure 4(a)), and celecoxib (15 *μ*mol/L) caused a reduction of 5.6-fold (Figure 4(a)). Similar results were obtained of cultures treated with drugs before infection for 1 hour, or when the drugs were added immediately after inoculation with virus.

### 3.4. Prostaglandin Synthetase Inhibitor-Induced HSV-1 Reduction Can Be Slightly Reversed by PGE_2_

To confirm the specificity of the decrease in virus yields by prostaglandin synthetase inhibitors, we investigated whether the addition of exogenous PGE_2_ could restore virus growth in the presence of COX inhibitors. At the indicated drug concentrations, there was a slightly increased yields of virus when both inhibitors and PGE_2_ were added compared to those obtained by adding the inhibitors alone, but this yield rarely reached control levels where no drugs were added (Figure 4(b)). This reversal effect was more obvious when PGE_2_ was used in combination with celecoxib. Moreover, PGE_2_ added alone at this concentration altered virus growth, with an increase of 2-3-fold in the production of infectious viral progeny (Figure 4(b)).

### 3.5. The Effects of Indomethacin on the Reactivation of HSV-1 in Latently Infected VGNs

**Figure 5:**
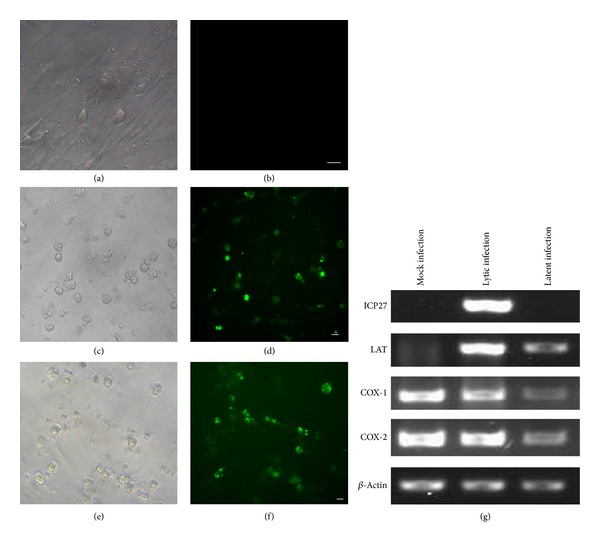
Latent HSV1 infection and reactivation in cultured VGNs. Light microscopy (a) and fluorescent microscopy (b) of latently infected VGN cultures. Light microscopy (c) and fluorescent microscopy (d) of latently infected VGN cultures treated with TSA. Light microscopy (e) and fluorescent microscopy (f) of latently infected VGN cultures treated with a combination of TSA and indomethacin. Detection of HSV-1 transcripts and COX-1/COX-2 mRNA by RT-PCR (g). Scale bar, 20 microns.

**Figure 6:**
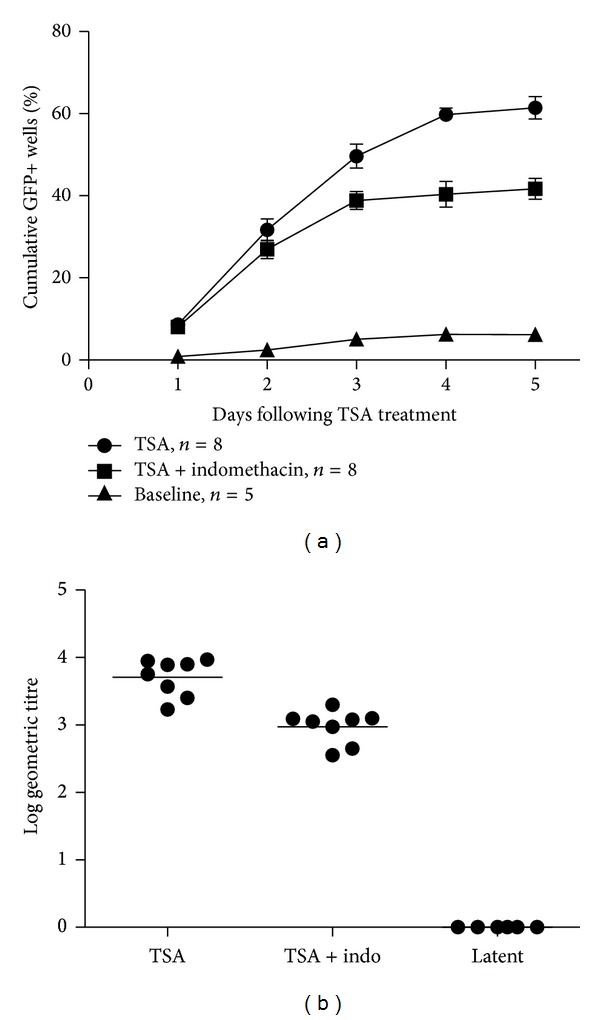
Effects of indomethacin on reactivation of HSV-1. (a) HSV1 reactivation was measured by the cumulative percentage of GFP-positive wells using fluorescence microscopy. Black triangles = baseline reactivation; black circles = TSA-treated; black squares = TSA- and indomethacin-treated. Error bars = standard error of the mean. (b) HSV-1 titer measured by TCID50 on media from latently infected (latent), latently infected TSA-treated (TSA), and latently infected TSA- and indomethacin-treated (TSA + indo) cultures. Viral titer = 0.7 TCID50. The *Y*-axis represents the log geometric titer.

We first performed RT-PCR and found that COX-1/COX-2 mRNA could be detected in both lytically and latently infected VGN cultures (Figure 5(g)). Following the establishment of latently infected VGN cultures (Figures 5(a) and 5(b)), RT-PCR demonstrated the presence of LAT (Figure 5(g)). And there was neither evidence of the lytic cycle ICP27 RNA (Figure 5(g)) nor infectious virus by TCID50 (Figure 6(b)). Treatment with TSA of latently infected neurons resulted in HSV-1 reactivation and GFP-positive neurons (Figures 5(c), 5(d), 5(e), and 5(f)). Reactivation was scored by the number of GFP-positive wells. Most of the GFP+ wells were detected within 5 days of addition of TSA. At later times, the reactivation rate remained relatively stable, and the time course of reactivation lasted 5 days to diminish the toxic effects of indomethacin on the neurons. We have done 8 independent experiments on different dates with neurons dissected from different animals. Each experiment consisted of an average of 11.7 wells of a 96-well plate as well as an average of 6 wells of baseline controls. TSA alone induced an average reactivation rate of 61.44% (±2.76%, *n* = 8) of latently infected VGNs. However, this rate was reduced to 41.71% (±2.55%, *n* = 8) when TSA was added in combination with indomethacin. During the first 2 days, there was no significant difference (*P* > 0.05, paired samples *t*-test) in reactivation rate between these two groups. However from day 3, *t*-tests showed statistically significant differences (*P* < 0.05, paired samples *t*-test) (Figure 6(a)). Reactivation of HSV-1 was confirmed by TCID50 of cells and supernatants from cultures at 5 days after TSA treatment. Results showed that infectious HSV-1 was produced in all of the cultures that showed GFP+ positivity (Figure 6(b)). We randomly titrated and compared 8 samples (3 wells/sample) from latently infected TSA-treated, and latently infected TSA- and indomethacin-treated wells. There was statistically significant differences (*P* < 0.05, independent-samples *t*-test) between them. Infectious virus progeny in the wells treated with indomethacin had an average of 5.86-fold reduction compared with those without indomethacin (Figure 6(b)).

## 4. Discussion

HSV-1 is an NT virus with a propensity to be taken up by sensory neurons and can assume a latent state in various types of ganglion cells, including TGs, geniculate ganglion, and VGs [20]. The COX-2 gene expression analysis in mouse TGs with latent HSV-1 indicated that the COX-2 gene expression was significantly upregulated after reactivation [21]. Several recent studies have emphasized the importance of COX in the mechanism of inflammation in the nervous system in experimental models. Pharmacological inhibition or genetic ablation of COX-1 activity decreased brain levels of PGE_2_, PGD_2_, PGF_2a_, and TXB_2_ and the expression of proinflammatory cytokines and chemokines after lipopolysaccharide injection [22]. Elevated levels of PGE_2_, PGF_2a_, and thromboxane B_2_ (TXB_2_) have been found in the cerebrospinal fluid of patients with HIV-associated dementia, indicating that COX may participate in the inflammatory response stimulated by viral infection in neural ganglia [23]. In our model, HSV-1 infection induced COX-2 production with an increase of 14-fold, which caused an increased production of PGs. PGs are important factors in the inflammatory cascade reaction. We assumed that if this happens to VG *in vivo* after HSV-1 infection, subsequent inflammation could bring severe damage to VGNs, which could serve as an explanation for VN or MD manifested as herpetic vestibular ganglionitis. VGN cultures in our studies were treated with 5-FU, aphidicolin, and serum starvation; thus, nonneuronal cells in these cultures remained viable but did not divide. The proportion of neural cells to nonneuronal cells was similar to that of the VG *in vivo*. COX-2 production under these conditions approximately represents the natural expression of this protein* in vivo*.

In this study, an *in vitro* cell culture system was set up for the first time to determine the effects of NSAIDs on the multiplication of HSV in VGN cultures. Previous experiments have suggested viral replication and reactivation could be interfered by modulation of PGs synthesis [15, 16]. Though the mechanism remains unknown, recent studies suggested PGE_2_ promoted viral replication via cAMP signaling pathway or during the stage of capsid assembly and viral maturation [16, 24]. However, drugs used in the present study showed limited inhibitory effects. Possible factors might take responsibility for this. One is that our culture system contains neurons and nonneuronal cells. Ideally, virus primarily infects the neurons and then spreads to the peripheral cells. Occasionally virus would primarily infect the nonneuronal cells; since the efficiency of viral infection of these two kinds of cells is much different, it could affect the ultimate results. Another most likely possible factor is that drug concentration in our experiments is severely restricted by its cytotoxicity. Our data indicated that the drug's inhibition effect showed a concentration-dependent feature. Concentration used in our experiments was too low to cause an ideal reduction in the treated group. While in the previous experiment which was done in fibroblast cell line [16], concentration of indomethacin used by the authors had a 2.5-fold increase compared with that used in our study, and this concentration could kill all the cells in our culture system (which we have tested). The last but not least is that cyclooxygenase may make a difference *in vivo*, in that this enzyme may take effects through a complete inflammatory cascade reaction which was not present in our culture system. Byproducts of inflammatory response other than PGs may play relatively major roles in the production or reactivation of HSV-1 *in vivo*. Thus, further studies are needed to confirm our results.

Animal models of HSV-1 latent infection in VG are difficult to construct due to the fact that VG are deeply imbedded in the temporal bone. Thus, VGN culture system was applied in our study. We used the TSA-induced reactivation protocol previously described in many systems *in vitro* [12, 25]. TSA has been shown to reactivate latent HSV1 infection by deacetylating viral DNA and allowing for the transcription of transcripts. Our data demonstrated that reactivation of HSV-1 in wells occurred within the first 5 days after induction. At later times, the reactivation rate remained relatively stable. Thus, the 5-day data presented in this study supported the conclusion. In our study, there was a tendency that the drugs affected HSV-1 reactivation rate from day three following application of TSA. Probably, during the first two days, deacetylating might play a predominant role in reactivating latent HSV-1. Subsequently, the presence of COX and its production of PGs might enhance this process. Inhibition effects of indomethacin on baseline reactivation had no statistically significant difference (data not shown), which might be due to the reason that spontaneous reactivation had relatively less correlation with the induction of COX. Although less is known regarding the mechanisms of this drug and its inhibition effects on viral reactivation, these results provided a stimulus for further investigation.

Taken together, Though our results are not so optimistic and further investigations *in vitro* or *in vivo* are required to confirm the effects of COX inhibitors, our study provides a potential way to investigate the mechanism of HSV-related vestibular pathogenesis as well as new pharmacotherapies in the treatment of intractable vertigo-related diseases.
